# Using machine-learning strategies to solve psychometric problems

**DOI:** 10.1038/s41598-022-23678-9

**Published:** 2022-11-07

**Authors:** Arthur Trognon, Youssouf Ismail Cherifi, Islem Habibi, Loïs Demange, Cécile Prudent

**Affiliations:** 1 Clinicog, 185 rue Gabriel Mouilleron, 54000 Nancy, France; 2 INSERM, CNRS, Institut de la Vision, Sorbonne Université, 17 Rue Moreau, 75012 Paris, France; 3 Lorraine University, 23 Boulevard Albert 1er, 54000 Nancy, France; 4 BePsyLab, Angers University, 5Bis Boulevard Lavoisier, 49045 Angers, France

**Keywords:** Psychology, Experimental models of disease

## Abstract

Validating scales for clinical use is a common procedure in medicine and psychology. Through the application of computational methods, we present a new strategy for estimating construct validity and criterion validity. XGBoost, Random Forest and Support-Vector machine learning algorithms were employed in order to make predictions based on the pattern of participants’ responses by systematically controlling computational experiments with artificial experiments whose results are guaranteed. According to these findings, these approaches are capable of achieving construct and criterion validity and therefore could provide an additional layer of evidence to traditional validation approaches. In particular, this study examined the extent to which measured items are inferable by theoretically related items, as well as the extent to which the information carried by a given construct can be translated into other theoretically compatible normative scales based on other constructs (thereby providing information about construct validity); as well as the replicability of clinical decision rules on several partitions (thereby providing information about criterion validity).

## Introduction

Validating scales to discriminate between clinical populations is a relatively common procedure in psychology and medicine. The current validity framework postulates that validity corresponds to the level of evidence and theoretical justification that supports the interpretation and use of scores given by a scale. Thus, it is not the scale itself that is formally validated, but rather the interpretation of the scores it generates^1–3^.

The concept of validity generally crosses three major aspects: construct validity, content validity, and criterion validity. Construct validity is a central concept in psychology that allows us to identify the extent to which the proposed test allows us to identify the construct being measured, is generally obtained by correlating the measure with a certain number of other measures and whose correlation patterns are theoretically predictable^4^. In contrast to construct validity, content validity allows us to verify the representativeness of the items on a given instrument with regard to the construct under study, and the degree to which this instrument has an appropriate sample of items for the measurement of the construct under study^5^. Finally, criterion validity aims to measure the performance predicted by the measurement tool. In particular, it is underpinned by predictive validity, which aims to verify the performance of the test with respect to the criterion taken as the object of study, for example by predicting a diagnosis from the set of variables (i.e. items) considered^6^.

The use of computational approaches, such as supervised machine learning, has shown spectacular results on a wide variety of numerical problems. Therefore, such strategies are becoming increasingly popular for analyzing data in numerous research fields, such as physics^7^, chemistry^8^, ecology^9^, and neuroscience^10,11^, where supervised machine-learning classification techniques have been applied to differentiate between a clinical population that is typically hard to identify (suicide ideators) and neurotypicals by capturing neural signatures of conceptual representations in fMRIs and following the presentation of valence-bearing words on a screen.

In this paper, we postulate that supervised machine-learning strategies may represent a promising avenue for solving psychometric problems, or, more generally, may allow for an additional layer of evidence in approaches to scale validity assessment. We thus present an analytical framework of computational psychometrics inspired by the rationale of experimental design in animal research^12^; and based on comparative readings of algorithmic predictions to control for overlapping information between certain groups of variables that could represent psychological constructs.

In general, supervised machine-learning methods are composed of two phases: a first phase that aims to create a model from a finite number of available data (i.e., observations), and then a second phase that aims to evaluate the predictive ability of the model by solving a practical task, such as estimating a probability density, or assigning a class to an observation, on a different sample than the one used to build the model^13^.

The framework of supervised computational psychometrics proposes to use the predictive abilities of models trained on questionnaire data to produce relative metrics to assess the extent to which the different groups of variables considered (e.g. one or multiple scales, factors, etc.) share information about a given construct.

In the present work, we aimed to achieve construct validity using supervised regression as regression allows one or several particular continuous variables to be approximated by a set of other variables that are correlated to it or them^13^. In the same way, we used supervised classification, an algorithmic technique that allows the categorization of objects, i.e. the assignment of a given class to each observation of a sample based on statistical data^14^ to achieve criterion validity.

Our hypotheses were investigated using archival data obtained from Lorraine University, which studied the construct of paranoia using Fenigstein & Vanable's Paranoia Scale, intended to identify paranoid thinking, defined by the original author as a thinking style characterized by exaggerated self-referential bias (such as suspicion, external locus of control, feelings of resentment and ill will, mistrust…) and influencing everyday life^14^. A cross-reference has been made by further experimenters between this scale and three other subscales from the Minnesota Multiphasic Personality Inventory 2 Restructured Form (MMPI-2-Rf), including the Restructured Clinical 6 (RC6-Paranoia scale), a measure of persecutive ideation (such as fear of harm to oneself or others)^15^; the Response Bias Scale (RBS-MMPI-2-Rf), designed to detect symptoms associated with cognitive response biases^16^, given that paranoid patients often present Bayesian reasoning biases producing noise in decision-making processes^17,18^; as well as the Neuroticism Revised Scale (NEGE-r-MMPI-2-Rf), which measures negative emotions such as anxiety, insecurity, preoccupation, as well as general tendency toward dramatization and negative anticipation^15^, given that paranoid patients often presents delusions of anticipation of a threat or negative future events^19^.

These archival data were used to conduct two experiments: we first assessed construct validity using XGBoost, Random Forest, and Support-Vector Machine regression in order to reconstruct the participants' responses matrix for Fenigstein & Vanable from the samples for the other scales (i.e., RC6, NEGE-r, and RBS), and examined how different models performed differently under different scales in this task. The criterion validity was then assessed using the XGBoost, Random Forest and Support-Vector classifiers, where the classical decision rule of MMPI-2-Rf was utilized to predict the psychometric label (psychotypic or paranoid), based upon the different scales available, while observing the differential prediction capabilities of the models.

For each of these experiments, control experiments were conducted. Control experiments ensure that the observed results are not random events, thus allowing for the distinction between signal and background noise^20^. In general, a positive control is a group that is known to definitely have an event when exposed to a stimulus; whereas the negative control is a variable where no response is expected when exposed to a given stimulus, thus allowing for a baseline value for background noise^21^. In the present study, we used the MMPI-2-Rf Paranoia scale as a positive control. Indeed, based on the same construct as the Fenigstein & Vanable scale, and given that and given that this last was constructed on the basis of the original MMPI scale^14^, it should be able to reconstruct the participants’ responses matrix on the Fenigstein & Vanable scale in the regression experiment. Similarly, given that the label of Paranoia is made on the basis of the MMPI-2-Rf T-scores, one would expect a ceiling effect to be reached when the label is predicted with this specific scale (i.e. the maximum performance that can be expected). More generally, this step will verify the possibility of the operation. In addition, we used a matrix of 20 random points generated in the interval 1–5 (as in the Fenigstein & Vanable scale) as negative controls. Indeed, these variables should not be able to reconstruct the participants’ responses matrix on the Fenigstein & Vanable scale, as these data are not informative about the paranoia construct nor providing the information necessary to correctly identify paranoid individuals in the sample. More generally, this step will allow us to note the impossibility of achieving accurate discrimination in the absence of sufficient information about the measured construct.

## Experiment 1: Performing construct validity of Fenigstein & Vanable (1992) scale using supervised regression

### Material and method

#### Subjects

Four hundred and seventy five not preselected adults (mean age = 25.33 years, SD = 11.22; male n = 145; females n = 330) from the general French population participated in this study. Full measures were available for all subjects.

All participants received detailed information about the study purpose and objectives, and provided online informed consent to participate in the study. All procedures were conducted in accordance with the Declaration of Helsinki and the study protocol was approved by the Institutional Review Board Commission Nationale de l'Informatique et des Libertés (registration n°2225110v0).

#### Psychometric materials

##### MMPI-2-RF

The Minnesota Multiphasic Personality Inventory-2-Restructured Form (MMPI-2-RF) is a standardized psychological instrument for comprehensive assessment of psychopathology and/or personality assessing the subject’s psychological dynamics (e.g., psychopathological trouble, behavioral tendencies…). We used the French version of this tool which consisted of 51 clinical scales including a scale for diagnosing paranoia (Restructured Clinical 6-RC6) by implying questions about auto-justice moral, interpersonal sensitivity and mistrust. This tool was used as a positive control, measuring the same construct as the Fenigstein & Vanable scale. We also used two clinical scales from the MMPI-2-Rf, the Responses Biases Scales (RBS) and the Neuroticism Scale (NEGE), to assess the construct validity of the Fenigstein & Vanable scale.

##### Fenigstein & Vanable (1992) scale

This Paranoia Scale was designed to detect paranoia-related symptoms in nonclinical populations examining paranoid thinking^14^. In this work, we translated this tool to French and used it to verify its construct validity.

#### Machine-learning models

The machine-learning approach consists of training a model with a subset of the provided dataset and then testing the trained model on an unseen subset. In this study, we used three different models to assess the differential predictive validity of participants' responses to the different clinical subscales of the MMPI-2-Rf in order to predict Fenigstein & Vanable participant’s response: XGBoost, Random Forest, and Support-Vector Machines. XGBoost algorithm is a scalable end-to-end tree boosting system which is aware about sparsity and weight quantile sketch for approximate tree learning)^22^. In other words, a XGBoost algorithm is a gradient boosting algorithm (i.e. an ensemble method that combines predictions from several models into only one by modelling the next predictor based on its predecessor’s errors) and that use classical decision trees as predictors (for a gentle introduction to XGBoost, see^23,24^). XGBoost systems currently appear to be the best compromise between performance and ease of use, which is why it won Kaggle's Higgs Boson Machine Learning Challenge, placing it in the models of choice for studying complex data, in particular for structured or tabular datasets^25^. From another hand, Random Forest is a “meta-estimator” coming from ensemble methods that fits a number of decision trees on several sub-samples of the dataset in parallel. It uses averaging of multiple decision trees to both improve its predictive accuracy and control for over-fitting^26–28^. In this study, only the number of generated trees and the loss function were set by experimenter, while the remaining hyperparameters were used as defaults (i.e. they were not explicitly set by us). The number of generated trees was set 1000, as suggested by Probst, Wright & Boulesteix^29^ and for the loss function, we used the “mean-squared error” (MSE). Finally, Support-Vector Machines for Regression (SVR) is an algorithm that produces predictions based on only a subset of the training data, ignoring data close to the model predictions^30^.

#### Computational psychometrics rationale for construct validity

Concerning construct validity, it is necessary in our own framework to show that the regression algorithm is able to capture the responses of participants with high accuracy and show that this operation is not feasible using another scale that does not share any dimension with the construct of interest.

### Procedure

1. Data were collected online through word of mouth and social media;
2. The obtained dataset was then split randomly to constitute an independent training set (n = 380) and a testing set (n = 95) using scikit-learn library in Python^31^;
3. XGBoost regressors were used to predict participants' responses to Fenigstein & Vanable Scale from one or several other scales under these specific conditions:
  - *Positive control* Predicting Fenigstein & Vanable participants’ responses on the basis of MMPI-2-Rf Paranoia (RC6) Scale. This scale was selected as a positive control since the Fenigstein & Vanable scale was originally constructed based on the MMPI^14^;
  - *Experimental condition 1* Predicting Fenigstein & Vanable participants’ responses on the basis of two scales (MMPI-2-Rf RBS + MMPI-2-Rf NEGE) that both carry information about Paranoia construct. Algorithms trained with this composition should perform close to the positive control;
  - *Experimental condition 2 and 3* Predicting Fenigstein & Vanable participants’ responses on the basis of one of two other MMPI-2-Rf scales (MMPI-2-Rf RBS or MMPI-2-Rf NEGE). Algorithms trained with this composition should perform close to the positive control as well, to a lesser extent than in experimental condition 1 ;
  - *Experimental condition 4*: Predicting Fenigstein & Vanable participants’ responses on the basis of two scales (MMPI-2-Rf RBS + MMPI-2-Rf NEGE) that both carry information about Paranoia construct, but with the same number of items as Fenigstein & Vanable scale (20-items), in order to control this parameter. The remaining items were selected randomly;
  - *Negative control* Predicting Fenigstein & Vanable participants’ responses on the basis of random data generated between 1 and 5 (as in Fenigstein & Vanable scale). These variables were selected as negative control since they shouldn’t carry information about the paranoia construct.
4. The Euclidean Distances (given the continuous nature of the data) between the participants' actual response matrix and the predicted response matrix were computed for all conditions;
5. An ANOVA test (Euclidean distance ~ prediction source) was performed to assess if the predictive abilities vary across all prediction sources, and Tukey post-hoc procedure was performed to analyze the interactions between variables.

All machine learning models were coded in Python 3.9 using functions from the scikit-learn library, and for statistical analysis we used R with R v3.6.2 as the main interpreter. Figures were produced using MATLAB R2022a and we used Windows 11 Pro as operating system.

### Results and discussion

**Figure 1:**
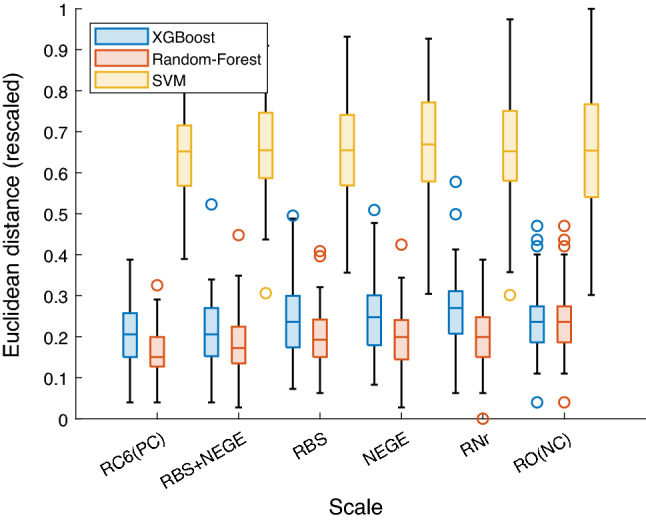
Mean distances between true and predicted matrices for prediction from RC6, RBS & NEGE (Combined), RBS, NEGE, RBS + NEGE (Reduced; RNr), and Random Observations (Negative control). Data were rescaled between 0 and 1.

Results of the Euclidean distance between real participant’s responses matrices and predicted ones can be observed in Fig. 1 and Table 1. Data suggest that only models trained with the RBS + NEGE composition, embedding two dimensions shared with the paranoia construct and with the completeness of their items, are able to reconstruct participants' response matrix to the Fenigstein & Vanable scale with the same performance as the RC6 scale, which specifically measures this construct.

**Table 1:** Post-hoc Tukey test results in regards of model performance to predict Fenigstein & Vanable participants’ responses matrix. [n.s.: not significant; RC6 (PC): Restructured Clinical 6 (Positive Control); NEGE: Neuroticism Scale; RBS: Responses Biases Scales; R + N(R): RBS + NEGE (Reduced); RO (NC): Random Observations (Negative Control)].

Scale	RC6 (PC)	RBS + NEGE	NEGE	RBS	R + N(R)	RO (NC)
RC6 (PC)	–	n.s	***p* < .001**	***p* = .02**	***p* < .001**	***p* < .001**
RBS + NEGE	n.s	–	n.s	n.s	n.s	***p* = .03**
NEGE	***p* < .001**	n.s	–	n.s	n.s	n.s
RBS	***p* = .02**	n.s	n.s	–	n.s	n.s
R + N(R)	***p* < .001**	n.s	n.s	n.s	–	n.s
RO (NC)	***p* < .001**	***p* = .03**	n.s	n.s	n.s	–

Significant values are in bolditalics.

Concerning the general performance measurements across the different algorithms, the ANOVA showed significantly different performances between them [*F*_*(2,1332)*_ = 4068.394, *p* < 0.001], without significant interaction with the experimental condition [*F*_*(10,1332)*_ = 1.306, *p* < 0.001].

Regarding specifically the Euclidean distance measures, predictions made using the combination of RBS and NEGE scales to predict the Fenigstein & Vanable scale produced the same results as in the positive control condition (RC6 to predict Fenigstein & Vanable, Table 1).

In contrast, predictions made using either of the scales sharing dimensions with the paranoia construct (i.e. RBS or NEGE) showed similar performance to the negative condition (i.e. Random Observation to Predict Fenigstein & Vanable, Table 1).

Interestingly, the RBS + NEGE composition controlled for number of items with the Fenigstein & Vanable showed statistically similar performance to both the positive and negative controls.

According to our observation, only the RBS + NEGE composition (two constructs which share dimensions with the paranoia construct as well as all of their items) acted as a positive control, whereas all of the other conditions acted negatively. Therefore, we can conclude that sufficient information about other constructs with which the construct under study shares dimensions is necessary for accurate estimation of participant response matrixes.

## Experiment 2: Performing criterion validity of Fenigstein & Vanable scale using random forest classifiers

### Material and method

#### Subjects

A subset (paranoiac: n = 131; controls: n = 131) of the initial sample was used for this experiment. We assumed that in classification study, experimenters would have initial knowledge about clinical conditions and would perform classification using two balanced groups.

#### Psychometric materials

##### MMPI-2-RF

WE used the same Restructured Clinical 6 (RC6) to test the criterion validity of the Fenigstein & Vanable scale.

##### Fenigstein & Vanable (1992) scale

WE used the same Paranoia Scale.

#### Machine-learning model

To assess if the Fenigstein & Vanable scale is sufficient to capture MMPI-2-Rf paranoia label, we constructed a second computational psychometrics experiment using the three models described above: XGBoost, Random Forest, and Support-Vector Machines. The classification performed in this study consists to predict MMPI-2-Rf paranoia diagnostic (i.e. T-score of MMPI-2-Rf Paranoia Scale > 80, as suggested in the MMPI-2-Rf manual^32^ and using a binary variable: 0 or 1) from the only basis of the participant’s responses to one given scale (excluding all other parameters such as age and gender that provides information about the subject). In this study, only the number of generated trees for Random Forest and the criterion were set by the experimenter, while all other parameters were set to default. The number of generated trees was set 1000, as suggested by Probst, Wright & Boulesteix^29^ and we used the “entropy” default criterion.

#### Computational psychometrics rationale for criterion validity

Concerning the criterion validity, it is necessary in our own framework to show that the classification algorithm is able to (1) capture the membership classes of the participants with a high sensitivity and specificity, (2) to show that this operation is feasible using other scales measuring dimensions shared with the construct of interest and (3) that this operation is not feasible in the opposite case.

#### Procedure

1. MMPI-2-Rf was scored automatically using a Python program to avoid human-error.
2. Normative data were encoded in Python to automate the transformation from raw data to T-score, then from T-score to diagnostic, according to MMPI-2-Rf cutoff^32^ (Paranoiac if T-score > 80). Descriptive statistics for these study groups are available in (Supplementary File 2: Supplementary Table 1).
3. Patients labeled "Paranoid" using the MMPI-2-Rf decision rule were assigned an age- and sex-matched control automatically using the R MatchIt function^33^.
4. The dataset was then split randomly to constitute an independent training set (n = 209) and test set (n = 53).
5. A k-Fold (with k = 10, according to Kohavi^34^) cross-validation was performed to assess the differential performance of the scales in automatically assigning participants' class from participants' responses to the respective scales, and accuracy, specificity, and sensitivity metrics were generated for all conditions:
  - *Positive control* Predicting MMPI-2-Rf label using participants’ responses on the basis of MMPI-2-Rf Paranoia (RC6) Scale. This scale was selected as a positive control as it produced the “Paranoia” label;
  - *Experimental condition 1* Predicting MMPI-2-Rf label using participants’ responses on the basis of participants’ responses on the Fenigstein & Vanable scale. Algorithms trained with this composition should perform close to the positive control as this scale was constructed using the original MMPI-2;
  - *Experimental condition 2* Predicting MMPI-2-Rf label on the basis of participants’ responses of the two other MMPI-2-Rf scales (MMPI-2-Rf RBS + MMPI-2-Rf NEGE) that both carry information about Paranoia construct. Algorithms trained with this composition should perform close to the positive control;
  - *Experimental condition 3 and 4* Predicting MMPI-2-Rf label the basis of participants’ responses on one of the other MMPI-2-Rf scales (MMPI-2-Rf RBS or MMPI-2-Rf NEGE). Algorithms trained with this composition should perform close to the positive control as well, to a lesser extent than in experimental condition 1;
  - *Experimental condition 5* Predicting Fenigstein & Vanable participants’ responses on the basis of two scales (MMPI-2-Rf RBS + MMPI-2-Rf NEGE) that both carry information about Paranoia construct, but with the same number of items as Fenigstein & Vanable scale (20-items), in order to control this parameter. The remaining items were selected randomly;
  - *Negative control* Predicting Fenigstein & Vanable participants’ responses on the basis of random data generated between 1 and 5 (as in Fenigstein & Vanable scale). These variables were selected as negative control since they shouldn’t carry information about the paranoia construct;
6. Two ANOVA experiments were performed in order to control clinical operating metrics:
  - Two-way ANOVA (accuracy ~ classifier*scale) was performed to assess the differential performance of the models on the “accuracy” general parameter;
  - Three-way ANOVA (score ~ classifier*scale*metric) was performed to control the performance of the scale in terms of sensitivity and specificity.
  - All significant interactions for (a) and (b) were controlled using a Tukey post hoc procedure.

All machine learning models were coded in Python 3.9 using functions from the scikit-learn library, and for statistical analysis we used R with R v3.6.2 as the main interpreter. Figures were produced using MATLAB R2022a and we used Windows 11 Pro as operating system. The ROC Curves for all these experiments are available to the reader in (Supplementary File 2, Figs. 1–3). We also performed a replication study by optimizing the hyperparameters by grid search and verifying that the models trained on the Fenigstein & Vanable scales behaved well like the models trained on the RBS + NEGE composition; in contrast to the models trained on age and gender on the one hand, or random points on the other hand, which should both behave similarly. The hyperparameter tuning is available in (Supplementary File 2: Table 2) and the differences between conditions for this additional experiment are presented in (Supplementary File 2: Table 3).

### Results and discussion

#### Accuracy

**Figure 2:**
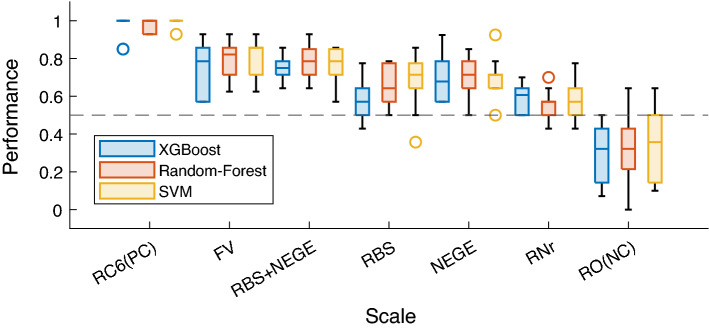
Results for the accuracy metric in tenfold cross-validation. [RC6 (PC): Restructured Clinical 6 (Positive Control); F&V: Fenigstein & Vanable; RNr: RBS + NEGE (Reduced); RO (NC): Random Observations (Negative Control); dashed line : randomness threshold].

Results for the accuracy measures are presented in the left panel of Fig. 2 and Table 2. Models showed varying performance when identifying participants' classes of membership based on the scales used to train the models, in contrast to the algorithms used for the experiments that did not appear to affect the models' performance.

**Table 2:** Tukey post-hoc test results for the accuracy. [RC6 (PC): Restructured Clinical 6 (Positive Control); F&V: Fenigstein & Vanable; R + N: RBS + NEGE (Combined); RO (NC): Random Observation (Negative Control)].

Scale	RC6 (PC)	F&V	R + N	RBS	NEGE	R + N(R)	RO (NC)
RC6 (PC)	–	***p* < *.001***	***p* < *.001***	***p* < *.001***	***p* < *.001***	***p* < *.001***	***p* < *.001***
F&V	***p* < *.001***	–	n.s	***p* < *.001***	***p* = *.03***	***p* < *.001***	***p* < *.001***
R + N	***p* < *.001***	n.s	–	***p* < *.001***	n.s	n.s	***p* < *.001***
RBS	***p* < *.001***	***p* < *.001***	***p* < *.001***	–	n.s	n.s	***p* < *.001***
NEGE	***p* < *.001***	***p* = *.03***	n.s	n.s	–	***p* < *.001***	***p* < *.001***
R + N(R)	***p* < *.001***	***p* < *.001***	n.s	n.s	***p* < *.001***	–	***p* < *.001***
RO (NC)	***p* < *.001***	***p* < *.001***	***p* < *.001***	***p* < *.001***	***p* < *.001***	***p* < *.001***	–

Significant values are in bolditalics.

Regarding the general accuracy measures, the different algorithms used showed similar performance [*F*_*(2,189)*_ = 1.954, *p* = 0.14], while the source scales to produce the MMPI-2-Rf label showed differential performance [*F*_*(6,189)*_ = 101.113, *p* < 0.001]. No interaction was detected between the scale used and the model used [*F*_*(12,189)*_ = 0.314, *p* = 0.31].

The results of post-hoc analysis revealed a significant difference in performance between all conditions (Table 2, Fig. 2 (Left)), with the positive control (RC6) exhibiting an extremely high accuracy (considered as the ceiling value since the diagnostic decision was initially based on a predefined cut-off from this scale). Moreover, under other conditions, analysis has shown that performances appear to decrease with the decrease in dimensions used, or the number of variables considered when generating predictions, and their performances are all distinguishable from the negative control despite being in between the two control conditions.

#### Detection metrics

Results for the clinical detection metric measures are presented in Table 3 and Fig. 2. The different scales showed differential performance in identifying patients, but similar performance in identifying control subjects, except for the RBS + NEGE (Reduced) condition.

**Table 3:** Tukey post-hoc test results for sensitivity and specificity metrics across prediction source. [RC6 (PC): Restructured Clinical 6 (Positive Control); F&V: Fenigstein & Vanable; R + N: RBS + NEGE (Combined); R: RBS; N: NEGE; R + N(R)/RNr: RBS + NEGE (Reduced); RO (NC): Random Observations (Negative Control)].

Metric	Sensitivity							Specificity						
Scale	RC6	F&V	R + N	R	N	RNr	RO(NC)	RC6	F&V	R + N	R	N	RNr	RO(NC)
RC6	–	***p* < .001**	***p* < .001**	***p* < .001**	***p* < .001**	***p* < .001**	***p* < .001**	–	***p* = .005**	***p* = *.003***	***p* < .001**	***p* < .001**	***p* < .001**	***p* < .001**
FV	***p* < .001**	–	*n.s*	***p* < .001**	*n.s*	***p* < .001**	***p* < .001**	***p* = .005**	–	*n.s*	*n.s*	*n.s*	***p* < .001**	***p* < .001**
R + N	***p* < .001**	*n.s*	–	***p* = *.02***	*n.s*	***p* = *.08***	***p* < .001**	***p* = *.003***	*n.s*	–	*n.s*	*n.s*	***p* < .001**	***p* < .001**
RBS	***p* < .001**	***p* < .001**	***p* = *.02***	***–***	*n.s*	*n.s*	***p* < .001**	***p* < .001**	*n.s*	*n.s*	*–*	*n.s*	*n.s*	***p* < .001**
NEGE	***p* < .001**	*n.s*	*n.s*	*n.s*	***–***	*n.s*	***p* < .001**	***p* < .001**	*n.s*	*n.s*	*n.s*	*–*	*n.s*	***p* < .001**
R + N(R)	***p* < .001**	***p* < .001**	***p* = *.08***	*n.s*	*n.s*	***–***	***p* < .001**	***p* < .001**	***p* < .001**	***p* < .001**	*n.s*	*n.s*	*–*	***p* < .001**
RO(NC)	***p* < .001**	***p* < .001**	***p* < .001**	***p* < .001**	***p* < .001**	***p* < .001**	–	***p* < .001**	***p* < .001**	***p* < .001**	***p* < .001**	***p* < .001**	***p* < .001**	–

Significant values are in bolditalics.

**Figure 3:**
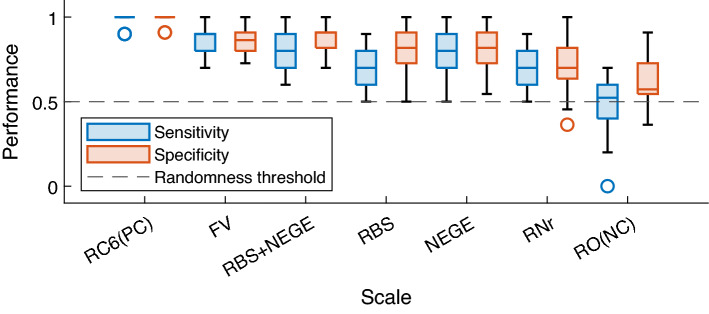
Results for the detection metrics in tenfold cross-validation. The model used was not considered as a variable given the absence of interaction of this variable with the other. [RC6 (PC): Restructured Clinical 6 (Positive Control); F&V: Fenigstein & Vanable; RNr: RBS + NEGE (Reduced) ; RO (NC): Random Observations (Negative Control); dashed line: randomness threshold].

Regarding the detection measures, the three-way ANOVA (score ~ classifer*scale*metric) revealed a significant effect of scale [*F*_*(6,378)*_ = 76.343, *p* < 0.001] and metric [*F*_*(6,378)*_ = 11.435, *p* < 0.001], with a significant interaction between the scale used to generate the prediction and the type of metric used to measure performance [*F*_*(6,378)*_ = 2.68, *p* = 0.01]. A subsequent post-hoc Tukey procedure revealed significant differences in sensitivity measures among all scales except those between F&V and R + N conditions (Table 3, Fig. 3), and NEGE when compared to the other positive conditions. Moreover, when the model was trained with a smaller number of items, F&V-based models performed significantly better than RBS + NEGE models (Table 3, Fig. 3), which is consistent with the results obtained in Experiment 1.

These results show that only the scales specifically measuring the paranoia construct or its equivalent in terms of information are able to correctly attribute the clinical condition to paranoid participants, whereas almost all are able to correctly identify control individuals. However, a significant difference was observed between the performance of classifiers trained on the RC6 scales and those trained on the Fenigstein & Vanable scales. This could be explained by the fact that the psychometric “Paranoia” label was obtained using the normative threshold of the MMPI-2-Rf, from which the RC6 scale is derived. As a result of this bias, the system should be able to perform the classification task to a greater degree. Nevertheless, this should not present a problem in future research, since clinical trials assign clinical conditions to participants based on medical diagnosis before the scales are administered.

Furthermore and interestingly, models trained on the basis of the NEGE scale alone show close performances to scales specifically measuring the paranoia construct in contrast to the other positive control scales, thus allowing inferences to be made about the informational content of the paranoia construct which seems to share much information with the neuroticism dimension.

## General discussion

Using supervised machine-learning approaches, we were able to perform two common validation operations in psychometrics, namely construct validity and criterion validity.

First, we performed a construct validation operation using state-of-the-art supervised regression algorithms (XGBoost, Random Forest, and Support-Vector Machines), showing that it was possible to reconstruct the participants’ responses matrix from responses to other questionnaires measuring the same constructs, or from responses to a set of questionnaires measuring constructs sharing dimensions with the construct of interest.

We then performed a predictive criterion validation operation using state-of-the-art supervised classification algorithms, showing that it was possible to automatically identify the class to which the participants belonged, in relation to two clinical groups (paranoid and neurotypical), using scales specifically measuring the construct of interest, or a set of scales sharing dimensions with it.

To our knowledge, and even though machine-learning paradigms have previously been employed to discriminate between different clinical groups using Likert scales and classification algorithms^35^, we believe that this work is the first to investigate the viability of these supervised approaches within the overall framework of the clinical scale validation approach. As such, it could achieve a proof of concept that computational approaches could be applied to solve psychometric problems, thereby providing an additional layer of evidence for validity when combined with traditional validation techniques.

Finally, we believe that beyond the framework of validation, and even beyond the general framework of the clinic, these types of approaches could have interesting perspectives in the research domains. Indeed, pre-trained classification models on clinical populations could perform automatic predictions on new and much more massive unlabeled datasets, and thus allow for large-scale multidimensional psychometric studies or even be used in clinical routine to quickly test a hunch based on a pre-trained model. In addition, these methods could be used to investigate in depth the extent to which certain dimensions are shared between certain constructs across different scales. In the case of the present work, it can be deduced from the ROC Curves (Supplementary File 2) that the majority contribution in the reconstruction of the paranoia construct was provided by the neuroticism scale. One could also imagine linking dimensions of different natures, for example by performing predictions of psychophysical data from cognitive and/or psychometric measures, thus allowing the creation of synthetic data sets to train theoretical models. Further research will therefore be needed to further evaluate the potential of these approaches in research settings and especially in terms of clinical translation possibilities.

However, the present study suffers from certain limitations. First, we were not able to control for the presence of corrupted or duplicate data, nor obtain complete information about the potential clinical categories of patients, given the fully computerized administration of the scales and the aleatory sampling for the collection of the data. Moreover, not all items of the MMPI-2-Rf were administered, thus not allowing for further convergence analysis. Finally, the study only covered two aspects of validation (i.e. construct and criterion validity), and not content validity. Further studies will therefore be necessary to evaluate the extent to which computational techniques could find their place within content validity demonstration approaches.

## Supplementary Information

Supplementary Information 1.

Supplementary Information 2.

## Data Availability

The dataset analysed during the current study is available in (Supplementary File 1).
