# Supplementary material for: Using machine-learning strategies to solve psychometric problems
Source: Sci Rep. 2022 Nov 7;12:18922. doi: 10.1038/s41598-022-23678-9 (PMC9640572; doi:10.1038/s41598-022-23678-9)
Supplement: Supplementary file 2 — Supplementary Information 2. [file 41598_2022_23678_MOESM2_ESM.docx]

**Using machine-learning strategies to solve psychometric problems**

**Arthur Trognon^1,3^, Youssouf Ismail Cherifi^2^, Islem Habibi^1^, Loïs Demange^3^, Cécile Prudent^4^**

*Supplementary Figures and Tables*


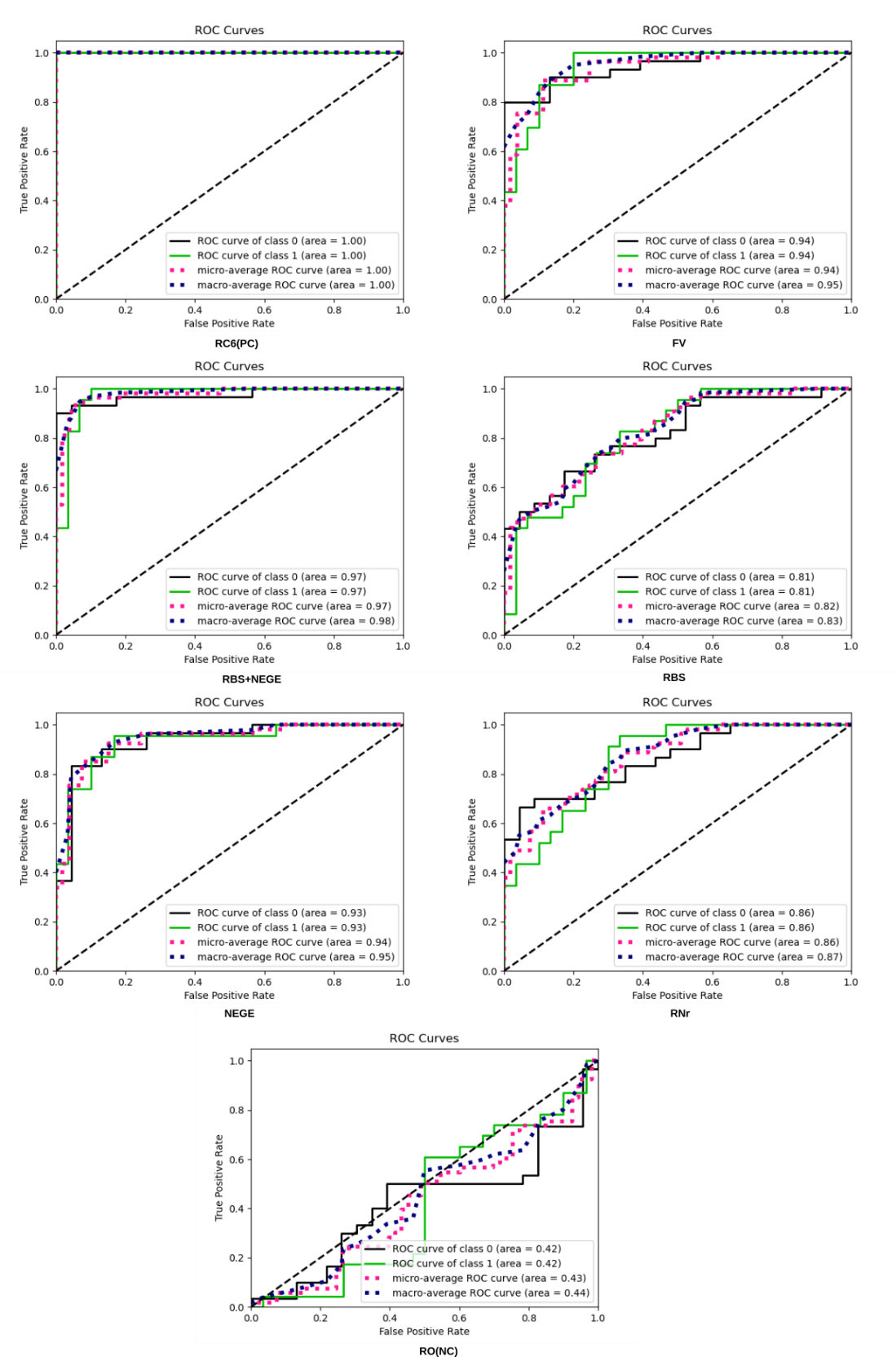


Supplementary Figure 1 : ROC Curves for the XGBoost models. RC6 (PC): Restructured Clinical 6 (Positive Control); F&V: Fenigstein & Vanable; RNr : RBS+NEGE (Reduced) ; RO (NC): Random Observations (Negative Control)


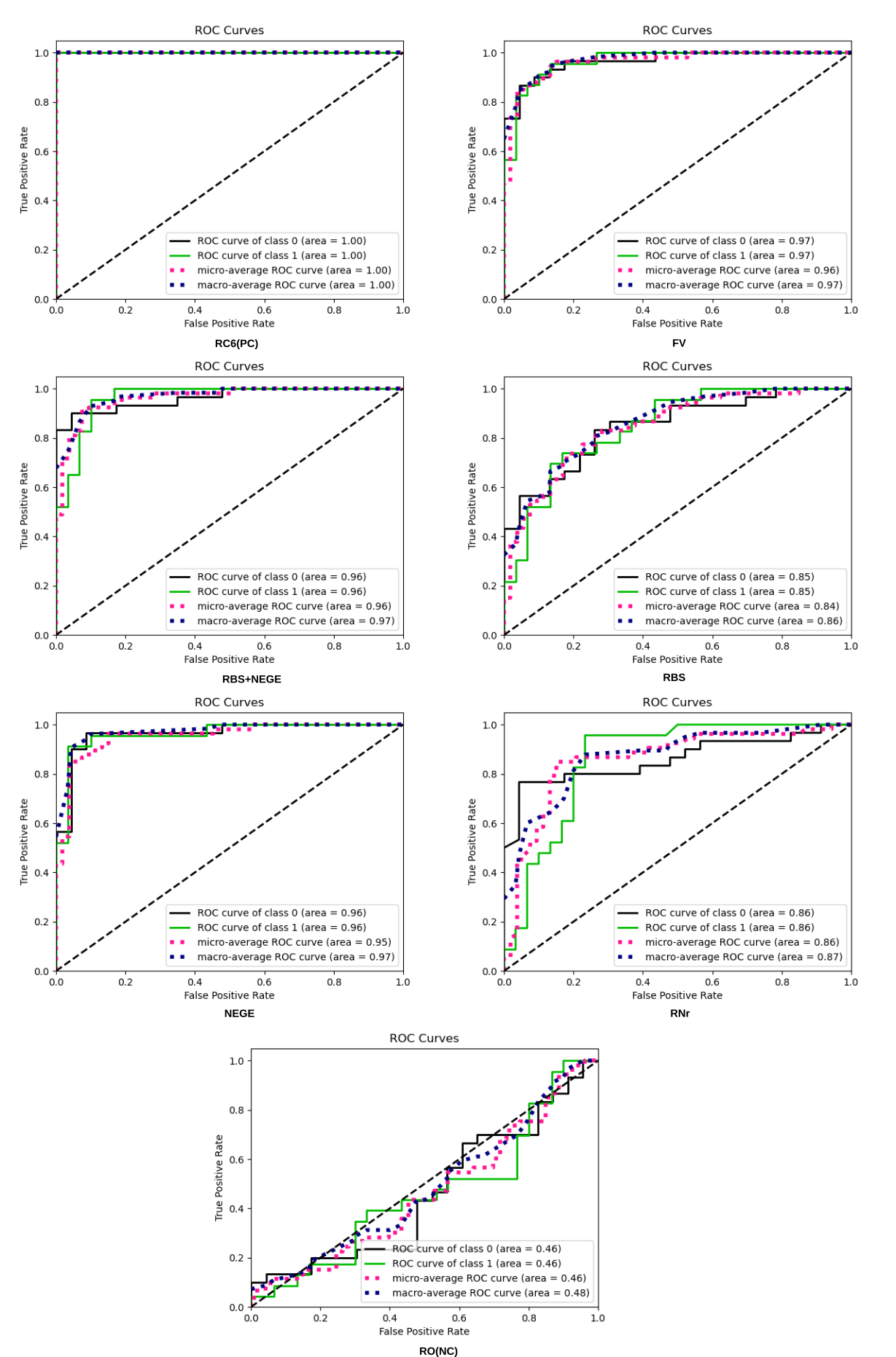


Supplementary Figure 2 : ROC Curves for the Random Forest models. RC6 (PC): Restructured Clinical 6 (Positive Control); F&V: Fenigstein & Vanable; RNr : RBS+NEGE (Reduced) ; RO (NC): Random Observations (Negative Control)


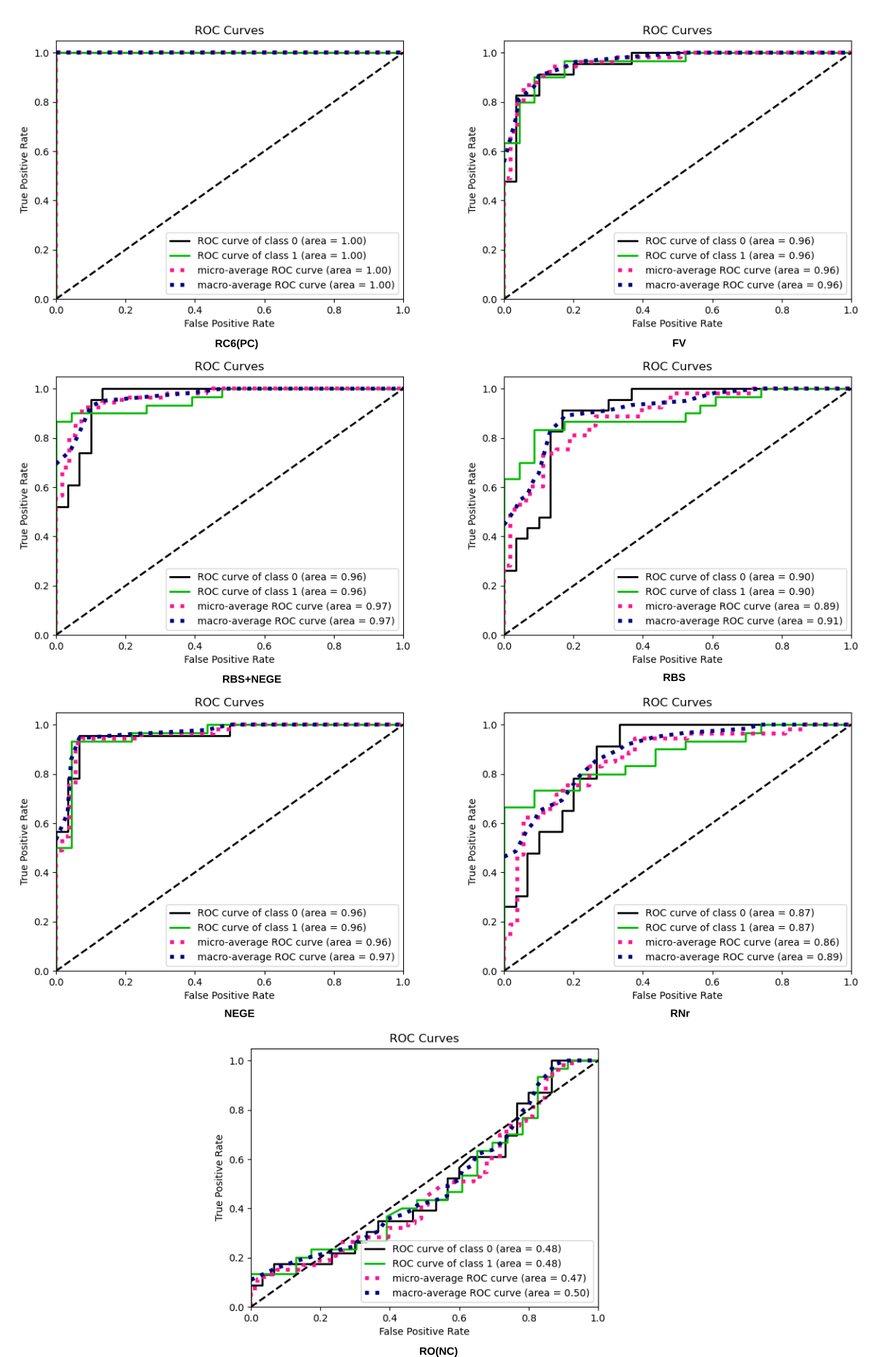


Supplementary Figure 3 : ROC Curves for the Support-Vector Machines models. RC6 (PC): Restructured Clinical 6 (Positive Control); F&V: Fenigstein & Vanable; RNr : RBS+NEGE (Reduced) ; RO (NC): Random Observations (Negative Control)

| Parameter | Control (n=131) | Paranoiac (n=131) | *p*-value |
| --- | --- | --- | --- |
| Age | 26.55(±12.52) | 24.21(±10.17) | .1 |
| Men | 50 | 25 | - |
| Women | 81 | 106 | - |
| Full scale (TP2S) | 14.63(±3.85) | 23.07(±4.2) | ***<.001*** |

Supplementary Table 1 : Descriptive statistics of the study sample for Experiment 2.

| Parameter | F&V | R+N (PC) | Age+Gender | Random Obs. |
| --- | --- | --- | --- | --- |
| Eta | 0 | 0 | 0 | 0 |
| Gamma | 0 | 0 | 0 | 0 |
| Max depth | 2 | 6 | 1 | 1 |
| Min. child weight | 1 | 0.5 | 0 | 1 |
| Max delta step | 0.1 | 2 | 1 | 1 |
| Subsample | 0.7 | 1 | 1 | 0.1 |
| Sampling method | Uniform | Uniform | Uniform | Uniform |
| Alpha | 0 | 0 | 0 | 0 |
| Lambda | 0 | 0 | 0 | 0 |
| Refresh leaf | 0 | 0 | 0 | 0 |
| Colsample (bytree) | 0.6 | 0.5 | 0 | 0.6 |
| Colsample (bylevel) | 0 | 0.6 | 0 | 0.5 |
| Colsample (bynode) | 0 | 0.6 | 0 | 1 |

Supplementary Table 2 : Grid search hyperparameter tuning for each classifier.

| Scale | F&V | R+N (PC) | A+G | RO (NC) |
| --- | --- | --- | --- | --- |
| F&V | - | .96 | ***<.001*** | ***<.001*** |
| R+N (PC) | .96 | - | ***<.001*** | ***<.001*** |
| A+G(NC) | ***<.001*** | ***<.001*** | - | .97 |
| RO (NC) | ***<.001*** | ***<.001*** | .97 | - |

Supplementary Table 3 : Tukey post-hoc test *p*-value results for the accuracy. [F&V : Fenigstein & Vanable ; R+N : RBS+NEGE (Combined) ; A+G (NC) : Age+Gender (Negative Control)]
